# Initial Ablation Ratio Predicts Volume Reduction and Retreatment After 5 Years From Radiofrequency Ablation of Benign Thyroid Nodules

**DOI:** 10.3389/fendo.2020.582550

**Published:** 2021-02-01

**Authors:** Stella Bernardi, Marco Cavallaro, Giacomo Colombin, Fabiola Giudici, Giulia Zuolo, Adrian Zdjelar, Chiara Dobrinja, Nicolò De Manzini, Fabrizio Zanconati, Maria Assunta Cova, Fulvio Stacul, Bruno Fabris

**Affiliations:** ^1^ Department of Medical Sciences, University of Trieste, Trieste, Italy; ^2^ Institute of Medicina Clinica, Ospedale di Cattinara, Azienda Sanitaria Universitaria Giuliano-Isontina, Trieste, Italy; ^3^ Unit of Radiology, Ospedale Maggiore, Azienda Sanitaria Universitaria Giuliano-Isontina, Trieste, Italy; ^4^ Unit of Biostatistics, Epidemiology and Public Health, Department of Cardiac, Thoracic, Vascular Sciences and Public Health, University of Padua, Padova, Italy; ^5^ Department of Radiology, Ospedale di Cattinara, Azienda Sanitaria Giuliano-Isontina, Trieste, Trieste, Italy; ^6^ Department of General Surgery, Ospedale di Cattinara, Azienda Sanitaria Giuliano-Isontina, Trieste, Trieste, Italy; ^7^ Department of Anatomical and Histo-pathology—Ospedale di Cattinara, Azienda Sanitaria Giuliano-Isontina, Trieste, Trieste, Italy

**Keywords:** radiofrequency ablation (RFA), initial ablation ratio (IAR), efficacy, regrowth, retreatment, 5 years, thyroid

## Abstract

**Background:**

Radiofrequency ablation (RFA) has gained ground as an effective and well-tolerated technique to treat benign thyroid nodules. Most of the available studies have described the short-term outcomes of RFA, whereas there is a limited number of studies evaluating long-term issues, such as regrowth and the likelihood of retreatments. In addition, risk markers of regrowth and retreatment remain to be defined. The initial ablation ratio (IAR) is an index that measures the amount of ablation after RFA, which has been associated with technique efficacy (i.e. volume reduction >50% after 1 year from the procedure). This study aimed at evaluating i) IAR reproducibility and ii) IAR predictive value for RFA 5-year outcomes.

**Materials and Methods:**

This is a retrospective single center study on patients with benign thyroid nodules treated with RFA and followed for 5 years after initial treatment. IAR interobserver reproducibility was evaluated with Bland-Altman method and Lin’s concordance correlation coefficient (ρc). IAR predictive value for RFA 5-year outcomes was evaluated with linear and logistic regression models, as well as with Cox models, while receiver operating characteristic (ROC) analyses were used for cut-offs.

**Results:**

We selected 78 patients with 82 benign thyroid nodules. The procedure significantly reduced nodule volume and this reduction was generally maintained over time. Technique efficacy was achieved in 92% of patients, while 23% of nodules regrew and 12% of nodules were retreated. Median IAR was 83%. Lin’s concordance and Pearson’s correlation coefficients suggested a good interobserver reproducibility of this index, consistent with the limits of agreement of the Bland-Altman plot. IAR was significantly associated with technique efficacy, 1- and 5-year volume reduction ratio, and with the likelihood of a retreatment, but not with nodule regrowth. ROC analyses showed that IAR cut-off was 49% for technique efficacy and 73% for retreatment.

**Conclusions:**

Our results show for the first time that IAR is reproducible and that it predicts the volume reduction and the likelihood of a retreatment after 5 years from RFA.

## Introduction

In the last decade, thermal ablation has gained ground as an effective treatment for symptomatic thyroid nodules (1, 2), particularly in patients refusing or having contraindications to standard treatment modalities, as well as in patients with recurrences of differentiated thyroid cancer (3). Thermal ablation refers to a group of techniques, whose operating principle is to induce nodule shrinkage by rapid heating and destruction of the target zone. These techniques include laser, radiofrequency, and microwave ablation, as well as high-intensity focused ultrasound (4). Among them, laser and radiofrequency ablation (RFA) are the most thoroughly assessed techniques.

Focusing on the use of RFA to treat benign thyroid nodules, this technique has been proven effective and well-tolerated. Several studies have demonstrated that RFA significantly reduces nodule volume, with improvement of local symptoms (5, 6). In addition, large retrospective series have demonstrated that RFA carries an extremely low risk (<1%) of major complications (recurrent laryngeal nerve injury or damage to cervical structures) (7), and that it does not impair thyroid function (8, 9), or subsequent thyroid surgery (10).

Most of the available studies have described the short-term outcomes of RFA, whereas there is a limited number of studies evaluating long-term issues, such as nodule regrowth and the likelihood of retreatments (11–13). We have recently shown that even though RFA induces a clinically significant and long-lasting volume reduction of benign thyroid nodules, 20% of patients experience nodule regrowth and 12% of patients are retreated over time (14). Nevertheless, risk markers of regrowth and retreatment are still limited and remain to be fully defined.

Recently, the ratio between the ablated volume and the total volume of a nodule, i.e. the initial ablation ratio (IAR), has been proposed as a semi-quantitative index that predicts technique efficacy and might predict long-term outcomes, such as regrowth and retreatment (15). In particular, the IAR was correlated with nodule reduction, and when the IAR exceeded 70%, nodules were reduced by more than 50% (15).

Thus, this retrospective study aimed at evaluating *i*) IAR reproducibility and *ii*) IAR predictive value for RFA 5-year outcomes, including regrowth and retreatment.

## Materials and Methods

### Study Design

This is a retrospective single center study, whose aims were to evaluate IAR reproducibility and IAR predictive value for 5-year outcomes of thyroid RFA, such as efficacy, volume reduction, regrowth and retreatment. For this purpose, we screened all the patients treated with thyroid RFA in the Hospitals of ASUGI (Azienda Sanitaria Universitaria Giuliano Isontina) in Trieste (Italy). Patient inclusion criteria were as follows: *i*) benign cytology before RFA (diagnostic category Thy2/Tir2 or Bethesda II, as assessed by FNAB and cytologic examination); *ii*) no prior thyroid treatment (radioiodine, ethanol injection); *iii*) follow-up of 5 years after the first ablation; and *iv*) availability of B-mode US scan images to calculate IAR. All patients were asked to give their written informed consent before inclusion. This study is part of the project 268_2019FYTNAB, whose protocol was approved by the local Ethics Committee (CEUR-2020-Os-039), and which was conducted in accordance with the declaration of Helsinki.

The following parameters were collected: age, sex, year of treatment, baseline nodule volume (ml), nodule structure, nodule function (non-functioning/autonomously functioning nodules), nodule volume (ml) after 1, 2, 3, 4, 5 years from the treatment. Technique efficacy, regrowth and retreatment were recorded as binary variables (yes/no). *Nodule volume* was measured by US examination, which was generally performed with linear transducers except for very large nodules, whose volume was quantified with convex transducers. *Volume reduction ratio* (VRR) was defined as the percentage reduction in volume and it was calculated as follows: VRR = [(initial volume – final volume)/initial volume] x 100. *Nodule structure* was classified as solid if the fluid component was ≤10%, predominantly solid if the fluid component was between 11%–50%, predominantly cystic if the fluid component was between 51%–90%, and cystic if the fluid component was >90%. *Nodule function* was assessed with laboratory examination as well as thyroid scintigraphy, which was performed in case TSH was <0.4 microU/ml. *Technique efficacy* was defined as a volume reduction ≥ 50% after 1 year from the treatment. *Regrowth* was defined as a ≥50% increase compared to the previous smallest volume at US examination (14, 16).

### Initial Ablation Ratio Measurement

**Figure 1:**
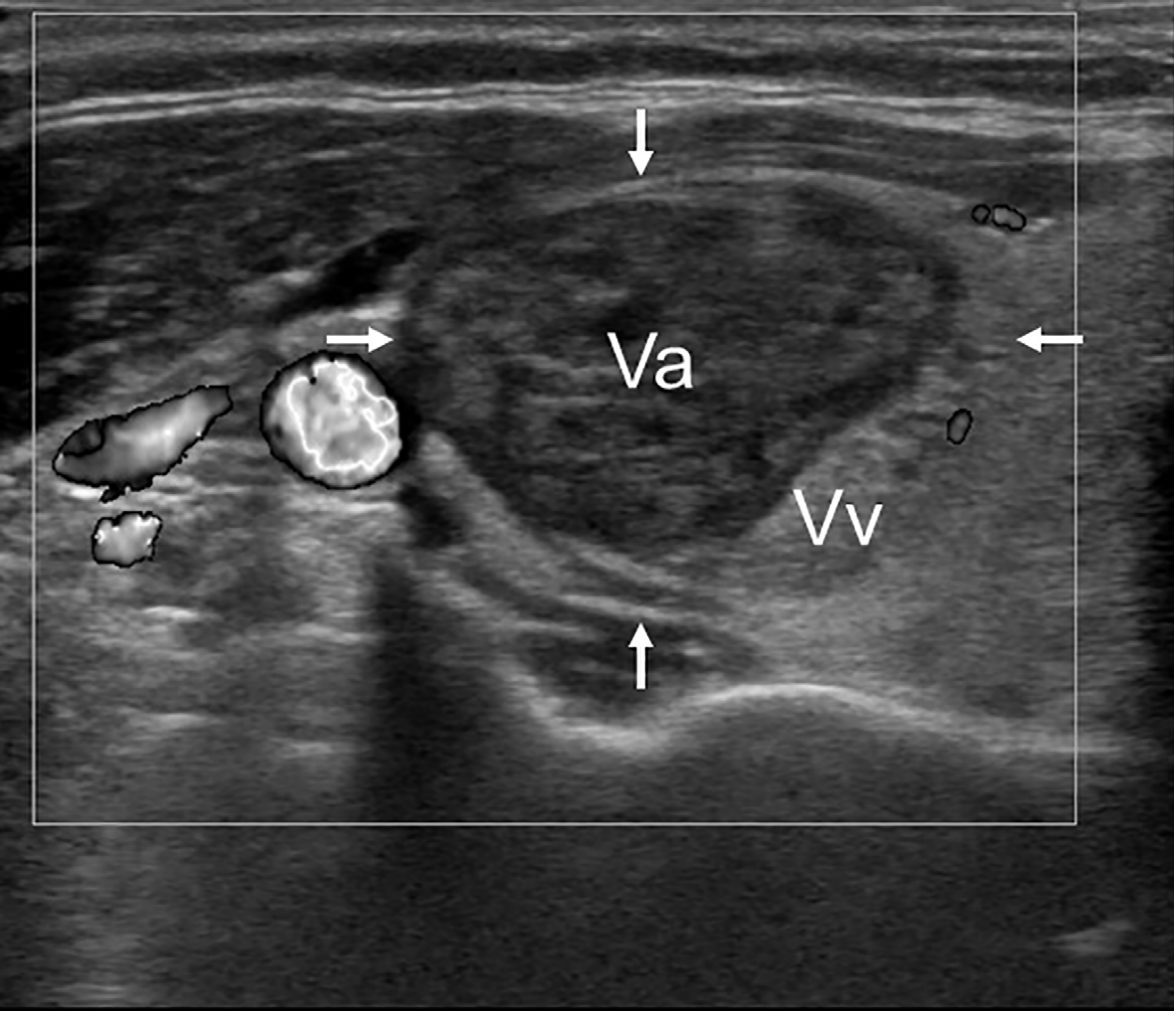
Representative B-mode US image of a thyroid nodule 1 month after RFA, showing that total volume (arrows) can be divided into an ablated volume (Va) and a vital volume (Vv). In particular, the ablated volume (which corresponds to the treated area) appears hypoechoic and avascular.

The initial ablation ratio (IAR) is a semi-quantitative index that measures the amount of ablation, which has been associated with technique efficacy (15). IAR measurement is based on the concept that the total volume (Vt) of a nodule can be divided into an ablated (Va) and a vital portion (Vv), i.e. Vt=Va+Vv, as shown in **Figure 1**. The IAR is the ratio between the ablated volume (Va) and the total volume (Vt), and it is calculated as follows: IAR=(Va/Vt) x 100. Of note, the Vt that should be taken into account is the one measured prior to RFA. In this study, in order to evaluate the interobserver variability of IAR measurement, IAR was assessed by two Radiologists (GC and AZ) on B-mode US scan images that had been recorded at baseline and 1-month after RFA. All the B-mode US scans were performed in the same Institute by two Radiologists (MC and FS). All RFA procedures were performed by one Radiologist (FS).

### Statistical Analyses

All statistical analyses were carried out in R system for statistical computing (Ver. 3.5.0; R Development Core Team, 2018). Statistical significance was set at p<0.05.

Shapiro-Wilk test was applied to quantitative (continuous) variables to check for distribution normality. Continuous variables were reported as median with range (minimum-maximum). Qualitative (categorical) variables were reported as absolute frequencies and/or percentages (rates of technique efficacy, regrowth and retreatment). Continuous variables were compared by student’s t test (and ANOVA) or by Mann–Whitney test (and Kruskall Wallis test), depending on data distribution and number of groups. Categorical variables were compared by Chi-square test or Fischer’s exact test whenever appropriate. Variations over time of nodules’ volume were evaluated with non-linear mixed-effects models (NLME) for repeated measures. Multiple comparisons of nodules’ volume with respect to different follow-up periods (baseline vs 1, 2, 3, 4, and 5 years) were performed with Friedman test for repeated measures and p-values adjusted with Bonferroni post-hoc test.

The interobserver variability of IAR was evaluated by assessing the agreement and reliability of IAR measurements performed by two operators. The Bland-Altman plot was used for analyzing the IAR interobserver agreement: the mean difference and the limits of agreement were calculated. In addition, IAR interobserver reliability was assessed with Lin’s concordance correlation coefficient (ρc) (17). This coefficient evaluates the reproducibility in measuring a continuous variable. In particular, ρc contains a measurement of precision (how close the data are about the line of best fit) and a measure of accuracy (how far the best-fit line deviates from the 45° line through the origin, this 45° line representing perfect agreement). This coefficient is used as a complementary approach to the Bland-Altman analysis. Like a correlation, ρc ranges from -1 to 1, with perfect agreement at 1 while values near to 0 indicates no agreement. The value of ρc can be interpreted with the Landis and Koch scale (0.2–0.4: fair; 0.4–0.6: moderate; 0.6–0.8: substantial; 0.8–1.0: almost perfect).

To evaluate if IAR was associated with nodule volume reduction, we conducted a univariate linear regression analysis. Statistically significant variables with a p value <0.10 on univariate analysis were then selected for multivariate linear regression analysis. To evaluate if IAR could be a risk marker of technique inefficacy, regrowth, and retreatment we conducted a univariate logistic regression analysis and calculated the odds ratio of IAR as well as of age, sex, baseline volume, nodule structure and function. Given the small numbers of technique inefficacy (n=7), regrowth (n=19), and retreatment (n=10), the multivariate logistic regression analysis could not be performed. Moreover, given that retreatment is a time-dependent occurrence, a univariate Cox proportional hazards regression model was performed to verify if IAR was associated to retreatments (results reported as Hazards Ratios (HR) with 95% confidence interval (95%CI).

Receiver operating characteristic (ROC) analyses were used to calculate the accuracy of IAR as predictor of technique efficacy and retreatment. Area under the (ROC) curves with 95% confidence interval, were interpreted according to Sweets criteria, and were used to identify a cut-off value of IAR that best predicted technique efficacy and retreatment. Specificity and sensitivity were also calculated (95% confidence interval, CI). The best possible cut-off point was defined as the highest Youden Index [(specificity + sensitivity) – 1 (R package “OptimalCutPoints”)]. DeLong method was used to test the statistical significance of the difference between the areas under the curve.

## Results

### Study Population

Inclusion criteria were met by 78 patients (82 benign thyroid nodules), who were selected for this study. All patients were treated between 2012 and 2015 with RFA, which was performed with the moving shot technique and a monopolar 18-G needle (18). **Table 1** shows the clinical and US characteristics of the study population. Median age was 60 years (18–86); there were 59 females (76%) and 19 males (24%). Median baseline thyroid nodule volume was 11.3 ml (0.44–54.6). Nodule structure was solid in 44% of cases, predominantly solid in 35% and predominantly cystic in 21% of cases. The majority of nodules were non-functioning (66%), while the remaining were autonomously functioning thyroid nodules (34%).

**Table 1:** Characteristics of study population.

**Number of patients**		78
**Age (years)**		59.5 (18–86)
**Sex**	**M** **F**	19 (24.4%) 59 (75.6%)
**Number of nodules**		82
**Baseline nodule volume (ml)**		11.3 (0.44-54.6)
**Nodule structure**	**Solid** **Predominantly solid** **Predominantly cystic/cystic**	36 (43.9%) 29 (35.4%) 17 (20.7%)
**Nodule function**	**Non-functioning nodules** **Autonomously functioning thyroid nodules**	54 (65.9%) 28 (34.1%)
**IAR (%)**		83.8 (-48.2; 100)
**Technique efficacy**	**Yes** **No**	75 (91.5%) 7 (8.5%)
**Regrowth**	**Yes** **No**	19 (23.2%) 63 (76.8%)
**Retreatment**	**Yes** **No**	10 (12.2%) 72 (87.8%)

Continuous variables are reported as median (min-max).

### Nodule Volume Reduction

Nodule volume was significantly reduced by RFA (p<0.001 for repeated measures). In particular, the nodules’ volume was 11.3 ml (0.5–54.6) at baseline, 2.8 ml (0.02–52.6) after 1 year from the procedure, 2.6 ml (0.008–59.9) after 2 years, 2.07 ml (0.001–70.23) after 3 years, 2.26 ml (0.001–25.02) after 4 years, and 2.29 ml (0.001–23.44) after 5 years. In other words, the volume decreased by 76%, 76%, 77%, 79%, and 79% at 1, 2, 3, 4, and 5 years after RFA. Predominantly cystic nodules were associated with greater volume reduction after 1 year, while volume reduction did not differ between predominantly cystic and predominantly solid or solid nodules after 5 years from the treatment (**Table 2**).

**Table 2:** Linear regression models for 1- and 5-year VRR.

		1-year volume reduction ratio					
		Univariate linear regression			Multivariate linear regression		
		Beta	SE	*p*-value	Beta	SE	*p*-value
**Age (years)**		-0.002	0.002	0.15	//		
**Sex**	Male	-0.07	0.05	0.21	//		
**Baseline volume (ml)**		-0.003	0.002	0.06	-0.001	0.001	0.27
**Nodule structure**	PC	0.14	0.06	0.01	0.11	0.04	0.007
	PS	0.01	0.05	0.82	0.04	0.03	0.29
**Nodule function**	Non-AFTN	0.06	0.05	0.21	//		
**IAR**		0.005	0.006	<0.001	0.004	0.0007	<0.001
		**5-year volume reduction ratio**					
		**Univariate linear regression**			**Multivariate linear regression**		
		**Beta**	**SE**	***p*-value**	**Beta**	**SE**	***p*-value**
**Age (years)**		-0.002	0.002	0.38	//		
**Sex**	Male	0.02	0.07	0.72	//		
**Baseline volume (ml)**		0.0001	0.002	0.95	//		
**Nodule structure**	PC	0.09	0.07	0.18			
	PS	0.02	0.06	0.80	//		
**Nodule function**	Non-AFTN	0.03	0.06	0.64			
**IAR**		0.005	0.001	<0.001	0.005	0.001	<0.001
**Therapeutic efficacy**	Yes	0.36	0.13	0.006	0.25	0.11	0.02

AFTN is for autonomously functioning thyroid nodules, PC is for predominantly cystic, and PS is for predominantly solid.

### Interobserver Reproducibility of Initial Ablation Ratio

**Figure 2:**
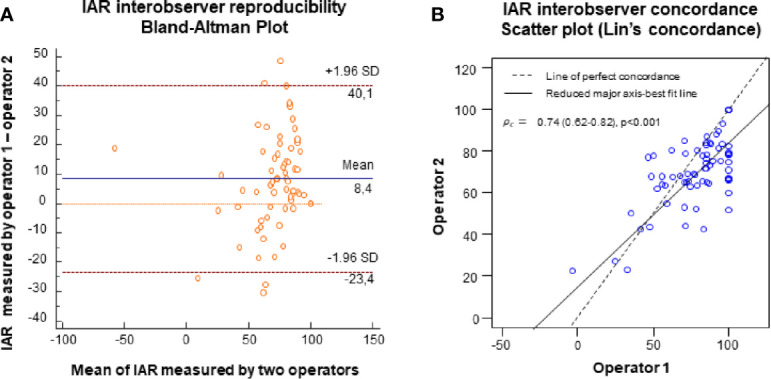
**(A)** Bland-Altman plot showing interobserver agreement of IAR measurements on B-mode US images. The x-axe shows the mean of IAR measurements, the y-axe shows the difference between the measurements. Orange line = 0; blue line = mean difference between operators, red lines = ± 95% ( ± 1.96 SD) limits of agreement. **(B)** Scatter plot of the Lin’s concordance (interobserver concordance) coefficient, showing how far the fitted relationship between x and y deviates from the 45° concordance line through the origin. The graph shows good precision (*ρ*=0.80), almost no systematic bias (*C*_b_), and a substantial interobserver concordance (*ρ*_c_=0.74).

Median IAR was 83% (-48%; 100%), as shown in **Table 1**. To measure IAR interobserver reproducibility and reliability, we compared the IAR values measured by two operators on the same nodules. The Bland-Altman analysis (**Figure 2A**) showed that the average discrepancy between the two operators was small, being 8.4 with 95% limits of agreement ranging between -23.4 and 40.1. Lin’s concordance correlation coefficient provided additional information, confirming a good interobserver reproducibility (**Figure 2B**). In particular, Pearson’s correlation (ρ) and Lin’s concordance (ρc) coefficients were respectively ρ=0.80 (95%CI; 0.68–0.86) and ρc=0.74 (95%CI: 0.62–0.82), indicating good reproducibility, with a bias corrector factor Cb of 0.94 (no systematic bias). It has been suggested that IAR reproducibility could further increase on contrast-enhanced US scans (19).

### Initial Ablation Ratio and Volume Reduction Ratio

**Figure 3:**
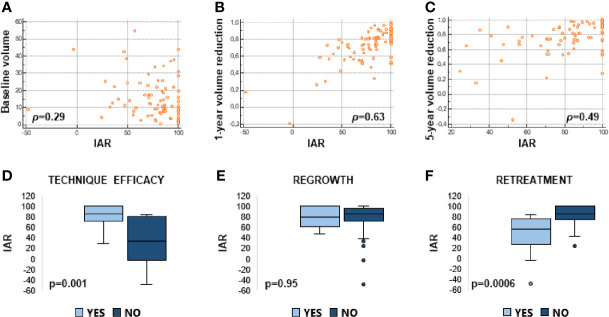
Linear correlations between IAR and baseline volume **(A)**, IAR and 1-year volume reduction ratio **(B)**, IAR and 5-year volume reduction ratio **(C)**. Box plots representing median IAR (min -max) in cases of technical efficacy vs inefficacy **(D)**; presence or absence of regrowth **(E)**; retreatment vs no retreatment **(F)**.

There was a good correlation between IAR and VRR after 1 and 5 years from the procedure (**Figures 3B–C**), whereas there was an inverse correlation between IAR and baseline volume (**Figure 3A**). On linear regression model analyses, IAR was independently associated with 1- and 5-year volume reduction (**Table 2**).

### Initial Ablation Ratio and Technique Efficacy, Regrowth, and Retreatment

Technique efficacy was achieved in 92% of nodules. Regrowth was observed in 23% of nodules. Retreatment was performed in 12% of nodules, as shown in **Table 1**. Causes of retreatment were: not reaching technique efficacy (40%), regrowth (40%), and symptom persistence (20%).

**Figure 3** shows that the amount of ablation (i.e. IAR) was significantly higher in the nodules that shrunk by more than 50% (**Figure 3D**) and that did not require any retreatment (**Figure 3E**), while IAR did not change between the nodules that regrew or not (**Figure 3F**). On logistic regression model analyses (**Table 3**), IAR was significantly associated with technique efficacy and the likelihood of not being retreated, but not with regrowth. Likewise, IAR was significantly associated with the likelihood of not being retreated over time as assessed by Cox regression model analysis (**Table 4**).

**Table 3:** Univariate logistic regression models for technique inefficacy, regrowth, and retreatment.

	Technique inefficacy(number of events = 7)		Regrowth(number of events = 19)		Retreatment(number of events = 10)	
	OR (95%CI)	*p*-value	OR (95%CI)	*p*-value	OR (95%CI)	*p*-value
**Age (years)**	1.03 (0.97–1.09)	0.29	0.96 (0.92–0.99)	0.03	0.97 (0.92–1.01)	0.14
**Sex** Female Male	1.00 (ref) 4.98 (1.00–24.7)	0.05	1.00 (ref) 0.50 (0.13–1.96)	0.32	1.00 (ref) 3.86 (0.98–15.2)	0.05
**Baseline volume (ml)**	1.02 (0.96–1.08)	0.54	0.97 (0.93–1.02)	0.30	0.97 (0.93–1.10)	0.10
**Nodule structure** S PS PC	1.00 (ref) 0.92 (0.19–4.50) 0.0002 (0.00–INF)	0.95 0.99	1.00 (ref) 1.59 (0.48–5.40) 2.73 (0.72–10.30)	0.47 0.14	1.00 (ref) 1.67 (0.40–6.88) 0.50 (0.05–4.85)	0.48 0.55
**Nodule function** AFTN Non-AFTN	1.00 (ref) 0.35 (0.07–1.70)	0.20	1.00 (ref) 2.31 (0.69–7.77)	0.18	1.00 (ref) 2.26 (0.45–11.50)	0.32
**IAR**	0.94 (0.90–0.98)	0.002	1.01 (0.98–1.03)	0.60	0.96 (0.93–0.98)	0.002

AFTN is for autonomously functioning thyroid nodules, PC is for predominantly cystic, PS is for predominantly solid, and S is for solid nodules.

**Table 4:** Cox proportional hazard regression model.

	Retreatment(n=10)	
	HR (95%CI)	*p*-value
**Age (years)**	0.97 (0.92–1.01)	0.13
**Sex** Female Male	1.00 (ref) 3.39 (0.99–11.73)	0.05
**Baseline volume (ml)**	1.04 (0.99–1.08)	0.09
**Nodule structure** S PS PC	1.00 (ref) 1.57 (0.42–5.85) 0.51 (0.06–4.54)	0.50 0.54
**Nodule function** AFTN Non-AFTN	1.00 (ref) 2.14 (0.45–10.1)	0.34
**VRR**	0.02 (0.004–0.13)	<0.001
**IAR**	0.97 (0.96–0.98)	<0.001

AFTN is for autonomously functioning thyroid nodules, PC is for predominantly cystic, PS is for predominantly solid, and S is for solid nodules, VRR is for volume reduction ratio.

### Initial Ablation Ratio Cut-Offs

**Figure 4:**
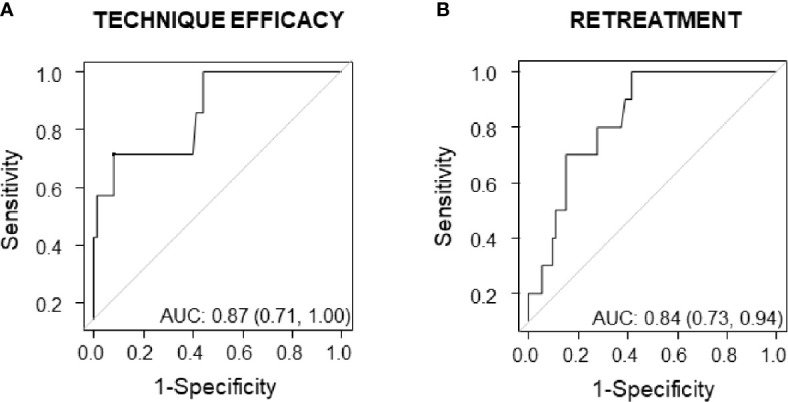
ROC curves showing predictive accuracy of IAR for technique efficacy **(A)** and for retreatment **(B)**.

On ROC curve analysis, when looking at technique efficacy, we found that IAR had an AUC of 0.87 (95%CI:0.71–1.00) and the cut-off best predicting technique efficacy was 49% (sensitivity = 0.71; specificity =0.92). When looking at retreatments, IAR had an AUC of 0.84 (95%CI: 0.73–0.94) and the cut-off best predicting no retreatment over time was 73% (sensitivity = 0.80; specificity =0.72). ROC curves are shown in **Figure 4**.

## Discussion

It has been largely demonstrated that RFA is an effective treatment for benign thyroid nodules. In randomized controlled trials, the volume reduction induced by RFA ranged from 69% to 78% after 1 year from the procedure (5, 6). Smaller baseline volume (20), spongiform US appearance (20), and higher amount of energy delivered (21) have been associated with this outcome. Consistent with these data, in this study, we found that volume reduction was 76% after 1 year from thyroid RFA and that it was significantly associated with nodule structure, as predominantly cystic nodules were reduced more than predominantly solid or solid nodules in the short term.

The IAR is a semi-quantitative index that measures the amount of ablation induced by radiofrequency. This index has been associated with technique efficacy (15). This study confirms that the amount of ablation, which was 83% was significantly associated with technique efficacy (i.e. 1-year VRR >50%), regardless of baseline volume and nodule structure. In particular, the IAR cut-off best-predicting technique efficacy was 49%. In other words, when more than 49% of the nodule is ablated, it is likely that the nodule will shrink by at least 50% after 1 year from the procedure. In addition, this study shows for the first time that IAR was significantly associated not only with 1-year but also with 5-year volume reduction.

As compared to the works by Sim (15) and Schiaffino (19), here we decided to ascertain if IAR measurement was reproducible. Our data show that IAR measurement, as assessed on B-mode US scans, was reproducible. In particular, despite the fact that the Bland-Altman plot showed that the 95% limits of agreement were wide for interobserver variability, the mean difference among observers was only 8.4%, suggesting that it is more likely that observers’ readings were closer to the reference measurement than to the extremes of the 95% limits of agreement. In addition, Lin’s concordance correlation coefficient was 0.74, indicating that there was substantial agreement.

It has been shown that thyroid RFA is effective in reducing nodule volume, and that the reduction achieved may remain stable for years. Nevertheless, in a minority of patients, regrowth may occur and patients may need a second treatment. To date, only a few papers have reported the 5-year outcomes of RFA. Most of them are limited by incomplete patient follow-up and variable number of treatment sessions (11–13, 22). In these studies, 5-year VRR ranged from 67% to 93.5%, whereas regrowth rate ranged from 4 to 24%, depending on the definition that was used. By contrast, in a study that followed a group of 216 patients for 5 consecutive years after thyroid RFA, nodule volume reduction was 77% at last follow-up, 20% of patients had nodule regrowth and 12% of patients underwent further treatments (14). In line with these figures, in this study, we found that after 5 years from RFA, nodule volume was reduced by 79%, 23% of nodules regrew, and 12% were retreated.

Unfortunately, the parameters that could help predict the risk of regrowth and/or retreatment over time remain to be fully identified (14). In a study evaluating the response of 206 nodules to RFA during a mean follow-up time of 22 months (range: 6–68 months), Yan showed that regrowth rate was 13% and that residual vital ratio, initial volume, location, and vascularity were all independent factors associated with regrowth (22). By contrast, in the study following 216 patients for 5 consecutive years after thyroid RFA, the only variable significantly associated with regrowth was the quantity of energy delivered, which turned out to be a poor predictor of regrowth, but a good predictor of retreatment (14). Consistent with these data, in this study, IAR was significantly associated with the likelihood of being retreated after 5 years from RFA but not with nodule regrowth. In particular, IAR cut-off best predicting no need of retreatments was 73%. In other words, when more than 73% of the nodule is ablated further treatments are unlikely. To translate it into clinical practice, an IAR value <73% might identify those patients who will benefit from a closer follow-up, as they are more likely to be retreated.

Our data suggest that predictors of technique efficacy and retreatment, such as IAR, are not necessarily predicting regrowth. The underlying reasons might include the fact that the current definition of regrowth is quite broad, and—for instance—it might fail to differentiate subclinical from clinical regrowths. Most importantly, it has to be taken into account that retreatments are due not only to regrowths but also to unsatisfactory volume reduction or symptom persistence, indicating that regrowth and retreatment are not exactly the two sides of the same coin.

Strengths and limitations of the study. The strengths of this study include the fact that it addresses for the first time the issue of IAR reproducibility and its predictive value for regrowth and retreatment over 5 years of follow-up. In addition, there was no patient loss during the follow-up, and being a single-center study we limited biases due to too many operators performing RFA and US scans. On the other hand, the limitations of this study include its retrospective design, and the low number of events (technique inefficacy, regrowths, and retreatments), such that we could not perform any logistic multivariate analysis.

In conclusion, this study shows that IAR is an index that is reproducible, and that it correlates not only with 1-year volume reduction and technique efficacy, but also with 5-year outcomes of RFA, such as 5-year volume reduction and the likelihood of being retreated over time. For this reason, the IAR is an index that might help clinicians in patient management. In particular, when more than 73% of the nodule is ablated, the need of further treatments is unlikely for—at least—5 years after thyroid RFA. Further studies with larger cohorts of patients are needed to confirm and extend our data, in order to identify markers of regrowth.

## Data Availability Statement

The raw data supporting the conclusions of this article will be made available by the authors, without undue reservation.

## Ethics Statement

The studies involving human participants were reviewed and approved by Comitato Etico Unico Regionale—FVG. The patients/participants provided their written informed consent to participate in this study.

## Author Contributions

SB, MC, and BF contributed to the conception and design of the study. MC and FS contributed to the data collection (they performed US scans) and FS performed all RFA procedures. GC and AZ contributed to the data collection (performed IAR measurements). FG performed the statistical analysis. SB, GC, and GZ organized the database. SB wrote the first draft of the manuscript. CD, NDM, FZ, and MAC contributed to patient recruitment. All authors contributed to the article and approved the submitted version.

## Conflict of Interest

The authors declare that the research was conducted in the absence of any commercial or financial relationships that could be construed as a potential conflict of interest.
